# Understanding the processes behind the decisions – GPs and complex multimorbidity decision making

**DOI:** 10.1186/s12875-022-01781-0

**Published:** 2022-06-28

**Authors:** Lloyd D. Hughes

**Affiliations:** Tayview Medical Practice Fife & NES Academic GP Fellow, University of St Andrews, St Andrews, Scotland

**Keywords:** Multimorbidity, Complexity, Decision-Making

## Abstract

Complex multimorbidity, defined either as three or more chronic conditions affecting three or more different body systems or by the patients General Practitioner (GPs), is associated with various adverse outcomes. Understanding how GPs reach decisions for this complex group of patients is currently under-researched, with potential implications for health systems and service delivery. Schuttner and colleagues, through a qualitative approach, reported that internal factors of individuals (decisions tailored to patients; Primary Care Physician (PCP) consultation style; care planning towards an agreed goal of care), external factors within the environment or context of encounter (patient access to healthcare; organizational structures acting as barriers), and relationship-based factors (collaborative care planning; decisions within a dynamic patient clinician relationship) all influence care planning decisions. There are other important findings which have broader relevance to the literature such as the ongoing separation of physical and mental health which persist even within integrated care systems, GPs continue to prioritize continuity of care and that organizational barriers are reported as factors in clinician decision-making for patients. More broadly, the work has proved valuable in extending previously reported findings surrounding care coordination, and limitation of current guidelines for patients with complex multimorbidity. Work-load in general practice is increasing due to an ageing population, increasing prevalence of multimorbidity and polypharmacy, and transfer of clinical activities from secondary to primary care. The future for GPs is more complexity in the clinic room, understanding how GPs make decisions and how this can be supported is crucial for the sustainability for general practice.

## Full paper

### Commentary

Multimorbidity, defined as the co-existence of two or more chronic conditions within an individual [1, 2], is now the norm in ageing populations [3]. Multimorbidity itself partially represents the success of chronic disease management and diagnostics, with patients with chronic physical diseases such as heart conditions, diabetes mellitus and obstructive respiratory diseases receiving significant improvements in medical and pharmacological interventions and associated outcomes over the last 20 years [4]. This commentary outlines the current state of multimorbidity and complexity from the perspective of General Practitioners, including the relevance of complexity, and provides further depth on a recent qualitative paper published in BMC Primary Care [5] on how physician clinical decision-making for complex patients with multimorbidity.

Multimorbidity provides significant challenges to the structure of healthcare services, which are often specialty or disease-focused in nature. There is considerable evidence suggesting that the current disease-based approach to managing patients with multimorbidity is associated with a variety of poor outcomes including inadequate preventative care and access to rehabilitation services [6], repeated referrals for specialist care [7] and increased healthcare costs [8]. The healthcare needs of patients with multimorbidity, particularly those in lower socio-economic groups and with mental health diagnoses, can be complex with different specialties focusing upon competing priorities (which may or may not be patient centered), demanding self-care regimes, polypharmacy and challenges in coordinating such care regimes [9]. Furthermore, there is work highlighting that patient**s** with multimorbidity can be considered are more at risk of adverse patient safety events [10], with mixed physical and mental health multimorbidity associated with the highest risk of patient safety incidents [11].

Multimorbidity, despite work looking at disease clustering, is generally accepted to be a heterogenous condition [4, 12]. This has significant implications for the interventions required to address the impact of multimorbidity on patients and healthcare providers, with single disease or symptom focused interventions unlikely to be particularly efficacious. It can be challenging for General Practitioners (GPs) within a busy clinical environment to find the terminology of multimorbidity particularly helpful presently. Indeed, many patients with multiple conditions are not complex particularly when concordant, and other patients with single diseases are complex. In some quantitative research, complex multimorbidity has been defined as three or more chronic conditions affecting three or more different body systems with data showing that such patients are more likely to receive specialist care compared to those with multimorbidity alone [13]. However, such measures continue operationally limited for GPs in the clinic. Indeed, the relationship between multimorbidity and complexity is not linear in terms of disease count, rather it reflects the challenges of delivering generalist care which is personalized to the patient (including biopsychosocial factors) alongside the interaction between individual conditions [14]. The individual GP is often well placed to recognize this complexity [15]. The CARE Plus Study was an exploratory cluster randomized controlled trial, which aimed to improve the quality of life in multimorbid patients living in areas of very high deprivation, and defined patients as complex based upon whether the GP felt the patient's health and biopsychosocial context, drawing on their experience, was complex rather than using questionnaires or an operation definition of complexity [15].

Why is complexity so pertinent to multimorbidity? Multimorbidity has been explicitly linked to complexity [16, 17]. Firstly, clinicians make decisions regarding therapeutic interventions despite an increasing mismatch between the ‘patient in the guideline’ and ‘patient in the clinic’ and often a limited evidence base for the patient sitting in front of them [4, 12]. Secondly, there is considerable recognition of the impact that socioeconomic factors have upon multimorbidity which can sometimes feel outside the control of GPs [18], including the development of multimorbidity at earlier age [19] and the risk of developing certain patterns of multimorbidity [20]. It is upon this background that understanding the clinical decision-making processes of GPs for patients with complex multimorbidity is becoming an increasing research area [21, 22], building on previous research which has reported different models of decision-making used by GPs for patients with multimorbidity [23] and decision-making tools for patients with multimorbidity [24]. A recent 2022 paper by Schuttner and colleagues has provided some timely insights relevant for primary care clinicians and researchers in this area, isolating the unique components of decision-making for complex multimorbidity such as competing disease and treatment interactions or psychosocial vulnerability [5].

In their paper Schuttner et al. sought to describe factors which affect primary care physicians (PCP) decision-making for more complex populations of patients, using a qualitative approach. The researchers interviewed 23 PCPs across the Veterans Health Administration (VHA) in America, using a 12-question interview guide to consider decision-making related to a recent encounter for a complex patient with chronic health conditions. Multimorbidity was defined in line with current agreed definition from the Academy of Medical Sciences [12], and importantly complexity was defined by the interviewed physician. Notably, the VHA is an integrated care system (ICS), an organizational structure providing an opportunity to improve access to healthcare, quality and continuity of clinical and healthcare services, alongside improving efficiency in the context of rising multimorbidity [5]. The authors reported that internal factors of individuals (decisions tailored to patients; PCP consultation style; care planning towards an agreed goal of care), external factors within the environment or context of encounter (patient access to healthcare; organizational structures acting as barriers), and relationship-based factors (collaborative care planning; decisions within a dynamic patient clinician relationship) all influence care planning decisions. These themes are clinically important when considered in the broader context of the delivery of care for patients with complex multimorbidity in primary care, with some particular findings worthy of particular discussion.

Firstly, the paper highlights that even within an ICS, specific aspects of care are felt to fall outside the scope of primary care. Mental health care provision is felt to be particularly siloed, affecting the ability of decision-making of PCPs. This finding is important for policy makers in other countries such as the United Kingdom (UK) where ICSs are being developed, and should help promote recommendations from the Royal College of Psychiatrists in the UK in order to improve the delivery of mental health services within ICS and accountable care organizations [25]. ICSs provide a real opportunity to improve and join up mental health services with the rest of the health and care system, but Schuttner et al. highlight that this does not happen automatically even in well-established ICS systems with implications for PCPs. Given that physical-mental health multimorbidity is associated with particularly poor outcomes for patients [26], further work to improve integration of mental health care provision is important to focus upon.

Secondly, patient access to healthcare is an important consideration for PCPs and influenced decisions to prioritize continuity of care and implement tools which aid the prioritization of patients likely to benefit from comprehensive needs reviews, such as the Care Assessment Need score which is a validated risk score used in the VHA predicting future hospitalization and/or mortality risk [27]. The fact that PCPs prioritize continuity of care is important, as continuity is commonly regarded as a metric of quality [28]. A nationwide study in Norway reported that longitudinal continuity (measured by visit patterns with different providers over time) was associated with startling improvements in emergency admissions and mortality with reductions of 25–30% [28]. British work focusing upon continuity for patients over 65 years old with dementia, reported that higher continuity in general practice care was associated with lower medication burden, reduction in delirium, and emergency admissions [29]. Continuity of general practice care is also associated with reduction in healthcare costs [30]. Relational continuity, an ongoing therapeutic relationship between a patient and care provider, can also help reduce ‘collusion of anonymity’ where a succession of clinicians focus on the immediate pressing problem [31]. This can be a particular challenge for patients with multimorbidity where there can be competing symptoms and conditions requiring input from clinicians and concerning patients [31]. Qualitative research from New Zealand looking at decision-making for patients with multimorbidity in primary care reported that an additive – sequential consultation model has been used by PCPs [22]. The approach refers to isolating patient priorities in order of importance from the patient and clinician perspective, before addressing them sequentially until the consultation time elapses. The remaining problems are deferred to another consultation, and again promotes continuity of care. Furthermore, regularity of scheduled GP contact has been suggested to mitigate the risk of hospitalization for chronic ambulatory care sensitive conditions, although the effect reduces as multimorbidity disease count increases [32]. In some insurance-based healthcare systems follow-up consultations may be financially penalized [22], but this approach may make delivery of primary health care more achievable by working through issues with patients over time.

 Thirdly, with organizational barriers reported by PCPs as part of the decision-making process for this group of patients efforts to identify what these are and how they may be addressed are of particular importance on a regional and national level. Dutch work reported considerable barriers to the delivery of patient-centered care, an important component of effective care for patients with multimorbidity [33], which were reported at the patient, organizational and national level [34] with a systematic review of qualitative research reporting similar challenges alongside the impact of fragmentation of healthcare and barriers to shared decision-making for PCPs when managing patients with multimorbidity [35]. The paper extends several other findings, such as the fact that care coordination is valued by primary care providers, the inadequacy of current guidelines for patients with multimorbidity, the benefits experienced by PCPs in collaborative team-based decision-making and that longer consultation times are prioritized by PCPs for patients with complex multimorbidity.

There are several limitations to the paper, in particular that it represents a sample from an American ICS so caution is required when extending the findings to a European healthcare system. More broadly, the lack of a clear consensus on the definition of complexity and subsequent reliance on subjective GP interpretation has challenges for ongoing research. Most available instruments measuring patient complexity to date include a variety of domains including health, social factors, service coordination and health literacy. The challenge here, similarly to multimorbidity and polypharmacy, is to develop a definition of operational utility for research whilst also holding some value operationally for clinicians. Efforts are being made in the area of complexity, with a Canadian study reporting that patient complexity by medical specialty, identified by 9 markers of complexity (number of comorbidities, presence of mental illness, number of physicians involved in each persons’ care, number of types of physicians in each patient’s care, number of prescribed medications, number of emergency department visits, rate of death, rate of hospitalization and rate of placement in long term care facilities), varied considerably [36]. Alongside increasing training in managing multimorbidity and complexity, which may be focused upon trainees in certain medical specialties managing patient with higher levels of complexity, the authors called for consideration to be given to weighting complexity for payment rather than fee for service where the type and duration of the contact is the primary driver of payment [36]. Although the study reported that GPs had the lowest level of complexity across the 9 markers, the challenge for family medicine is that when faced with patients with high levels of complexity they must work within shorter consultations with more limited access to investigations.

There is an extensive array of different interventions for patients with multimorbidity, with a varying evidence base. With an ageing population, and associated increases in chronic disease rates, the prevalence and incidence of multimorbidity and complex multimorbidity are likely to increase in the years ahead. The impact of this will be felt across the health system, but particularly primary care which is the most accessible and under-resourced [37]. There is no doubt that future for GPs is complex as we balance the demands of delivering high-quality safe patient care for a patient population with greater complexity, and as such understanding how as clinicians we can make effective decisions for our patients remains a pertinent area of future research endeavors. 

## Data Availability

Not applicable for commentary piece.

## Abbreviations

GPs: General Practitioners
PCPs: Primary Care Physicians
VHA: Veterans Health Administration
ICS: Integrated Care System
UK: United Kingdom

## Declarations

### Competing of interests

LDH is a member of the editorial board for the BMC Primary Care journal.
